# Assessing changes in ultraviolet radiation-exposed mouse skin using optical coherence tomography

**DOI:** 10.1371/journal.pone.0328647

**Published:** 2025-08-11

**Authors:** Anna V. Vejlsby, Celina Pihl, Rozarin Delal Kara, Merete Haedersdal, Peter E. Andersen, Catharina M. Lerche, Gavrielle R. Untracht

**Affiliations:** 1 Technical University of Denmark, Department of Health Technology, Kongens Lyngby, Denmark; 2 Department of Dermatology, Copenhagen University Hospital, Bispebjerg, Copenhagen, Denmark; 3 Department of Clinical Medicine, Faculty of Health and Medical Sciences, University of Copenhagen, Copenhagen, Denmark; 4 Department of Pharmacy, University of Copenhagen, Copenhagen, Denmark; University of Science and Technology of China, CHINA

## Abstract

Skin cancer is one of the most common types of cancer, frequently resulting from excessive exposure to ultraviolet radiation (UVR). Early detection and effective prevention of photodamage are important to mitigate the long-term risks of skin cancer. In this study, we investigate the use of optical coherence tomography (OCT) for assessing photodamage over time and the efficacy of photodamage prevention treatments non-invasively. Of the *n* = 81 hairless mice, 75 of them were exposed to ultraviolet radiation (UVR) three times per week to induce photodamage. Two different systemic photodamage prevention treatments were tested: nicotinamide mononucleotide (NMN) and polypodium leucotomos (PL). OCT images were acquired monthly on the back, side, and stomach of the mice over 7 months. Two OCT-derived metrics, skin thickness and the attenuation coefficient, $\mu_{OCT}$, were quantified using a custom-developed algorithm to evaluate photodamage. Significant differences in skin thickness (*p* = 0.038) and attenuation coefficient (*p*<0.001) were observed between the UVR control group and the non-irradiated control group after 7 months at the back. At month 7, no significant difference was observed in the attenuation coefficient between the UVR control group and the UVR + PL group, however, a significant difference was observed between the UVR control group and the UVR + NMN group. Additionally, our OCT-derived skin thickness was well correlated with the skin thickness measured from histology, demonstrating a strong alignment with these invasive ground truth findings. Altogether, our results indicate that OCT could be a useful tool for non-invasive monitoring of photodamage in skin and for monitoring the efficacy of photodamage prevention treatments.

## Introduction

Cutaneous squamous cell carcinoma (SCC) is one of the most prevalent types of skin cancer worldwide [1, 2]. More than 1 million new cases are diagnosed each year in the U.S. alone, particularly among fair-skin individuals. The incidence continues to rise annually, with high-risk patients such as immunosuppressed patients or those with a history of skin cancers facing a greater risk of SCC becoming aggressive and potentially fatal [3, 4]. The development of SCCs is largely attributed to increased exposure to ultraviolet radiation (UVR). Excessive exposure to UVR causes damage to the skin as epidermal cells absorb the majority of harmful UV rays [5]. In more severe cases, this damage can lead to skin cancer, including SCC [6]. When absorbed in the cells, UVR triggers a cascade of harmful effects, including DNA damage, oxidative stress, inflammation, immunosuppression, and altered signal transduction pathways, all of which contribute to carcinogenesis [5]. While surgical excision remains the primary treatment for low-risk cases, managing high-risk SCC and assessing photodamage to predict cancer risk is more complex and lacks standardized protocols [4].

Due to the consequences of UVR exposure, early detection and effective prevention of photodamage are key to reducing the incidence of skin cancer. Sunscreen is one of the most commonly used methods for sun protection, but it is often applied insufficiently, leading to inadequate protection [6]. Although there is currently no standardized method for non-invasively assessing photodamage before tumor onset, previous studies have shown that acute responses to UVR include epidermal thickening due to the proliferation of keratinocytes as part of the skin’s defense mechanism [7]. Today, the standard diagnostic approach for skin cancer relies on clinical evaluation followed by a biopsy and histopathological analysis [8, 9]. While this method is reliable, it is invasive, leaves scarring, and limits clinicians’ ability to take multiple biopsies from areas with numerous suspicious lesions. Given the limitations of the current diagnostic method and lack of prevention, there is a need for new, non-invasive, and in vivo approaches that can assess photodamage early and over time, as well as better ways to enhance prevention.

The challenge of reducing skin cancer prevalence can be approached in two ways: first, through the development of novel photodamage prevention techniques that are user-friendly, and second, through the development of screening tools for better assessment of photodamage and prediction of cancer risk. One promising approach for innovative photodamage prevention is enhancing the body’s natural defense through the use of oral photo-protective agents, such as polypodium leucotomos (PL) [10, 11], and nicotinamide mononucleotide (NMN) [12, 13]. Recent studies have explored their role in delaying or preventing the initiation of carcinogenic pathways [3, 5, 10], where both agents have shown potential in mitigating photodamage. Previous research has demonstrated that oral administration of PL can significantly delay tumor onset and reduce the incidence of actinic keratoses in hairless mice following UVR exposure [5]. In studies involving healthy volunteers, the combination of oral and topical PL has been shown to increase the minimal erythema dose, indicating enhanced protection against UV damage. PL is known for increasing the activity of antioxidant enzymes, boosting the tumor suppressor gene p53, and for anti-inflammatory properties [5, 14, 15]. The use of NMN has been shown to increase nicotinamide adenine dinucleotide (NAD^ + ^) levels [16]. NAD^ + ^ supports DNA repair pathways that are also believed to be involved in the repair of UVR-induced DNA damage, therefore NMN may also act as a photo-protective agent [17].

Another promising approach to reducing the prevalence of skin cancer is the development of novel screening tools to better assess photodamage and predict cancer risk so that preventative action can be taken in due time. Advancements in diagnostic tools like optical coherence tomography (OCT) may offer a non-invasive method to detect early signs of photodamage. OCT provides the ability to assess skin tissue at high resolution in vivo, using infrared light to capture cross-sectional images of the skin [18]. It potentially provides the required diagnostic information non-invasively [19] and has already been utilized to investigate photodamage in human skin [20, 21]. A promising aspect of OCT is the possibility to extract the attenuation coefficient $\mu_{\text{OCT}}$, a tissue parameter that quantifies the rate light is attenuated when passing through a medium [22]. The attenuation represents the combined effect of light absorbed and light scattered when propagating through the tissue. By extracting and analyzing the attenuation coefficient, it is possible to assess subtle changes in tissue that may serve as early indicators of photodamage, such as hyperkeratosis and alterations in cell morphology. This capability allows OCT to detect microstructural changes before they are visible, offering a more detailed and earlier assessment of tissue health. Other studies have shown that there are chemical and structural differences in cancerous tissue compared to surrounding healthy tissue [23, 24]. These differences impact tissue properties, and it has been demonstrated that OCT can detect variations in these properties. Other studies have employed the attenuation coefficient to distinguish healthy tissue, from diseased [25–29]. Turani *et al*. extracted the optical radiomic signatures from human skin tissue to detect malignant melanomas and were able to differentiate benign moles from melanoma with 97% sensitivity and 98% specificity [26]. Given OCT’s ability to detect subtle changes in tissue, OCT shows potential not only for identifying photodamage before tumors develop but also for monitoring the efficacy of photoprotective treatments over time.

In this study, we investigated whether OCT can be used to evaluate UVR-related changes in the skin over time. Further, we assessed whether OCT could be used to monitor the efficacy of PL and NMN as photo-protectants. Using the OCT-derived metrics: the attenuation coefficient and the skin thickness, we showed that we could measure significant changes in the skin in response to UVR. While most metrics indicated that PL and NMN did not significantly reduce photodamage, clear trends in the OCT-derived metrics indicate that OCT may detect subtle UVR-induced changes in the skin. These results indicate that OCT could be a useful tool for non-invasive monitoring of photodamage in the skin and for monitoring the effects of using photo-protectants.

## Materials and methods

### Animal model

This study included a total of 81 ${C3.Cg.Hr}^{hr}/TifBomTac$ hairless mice (Taconic, Ry, Denmark) aged 16 to 32 weeks at the start. Of the 81 mice, 75 of them were divided into three groups irradiated with UVR (UVR + NMN, UVR + PL, and UVR control), while the remaining six were in the control group. The mice were group-housed in separate polycarbonate cages containing dust-free sawdust bedding. All mice had all-day access to water and standard laboratory food (Altromin 1324 maintenance feed, Altromin Spezialfutter GmbH & Co. KG, Lage, Germany). The room temperature was maintained between 23-24^°^C and the mice were housed under a 12-hour light/12-hour dark cycle.

This study was approved by the Danish Animal Experiments Inspectorate (permit number: 2021-15-0201-00905) and undertaken at Bispebjerg Hospital from October 2023 to April 2024. All methods reported are in accordance with ARRIVE guidelines. Mice were monitored for humane endpoints including a sustained weight loss of 20% or more compared to an untreated control group of the same age and sex, persistent asocial or self-isolating behavior or lack of activity, flattened ears or hunched posture, and pale skin or grayish discoloration. Ruffled fur and squinted eyes were not applicable to this hairless mouse model. Additionally, we monitored for tumor-specific endpoints, including tumor location impairing normal bodily functions and tumor burden limited to a maximum of one tumor measuring 12 mm in diameter or three tumors measuring 4 mm in diameter. Mice were euthanized using either cervical dislocation or exposure to an 80/20 ${\text{CO}}_{2}/{\text{O}}_{2}$ atmosphere, in accordance with ethical guidelines. The entire experiment lasted a maximum of eight months and the mice were observed at least once daily. Our study was designed according to the 3Rs principles (Replacement, Reduction, and Refinement). The mice were anesthetized during the tattoo procedure at the beginning of the study and during subsequent imaging, as described below.

During the study, 14 mice died due to a predisposed phenotype; none of these deaths were related to the study. The death of an animal was not a planned outcome or experimental endpoint in our study. The ${C3.Cg.Hr}^{hr}/TifBomTac$ strain has an increased risk of spontaneous mortality, and previous examinations have reported acute circulatory stasis of major organs (lungs, liver, and kidneys) without signs of inflammation or tumor development. The spontaneous deaths occurred regardless of daily health monitoring with no prior clinical signs that could have enabled early intervention.

### Study design

This study was part of a larger investigation testing the drug efficacy of the two systemic photodamage prevention treatments: nicotinamide mononucleotide and polypodium leucotomos. From each of the four groups, six mice were selected for monthly imaging with OCT, forming four smaller subgroups. These subgroups included a control group, a UVR control group (exposed to UVR but receiving no systemic treatment), a group treated with nicotinamide mononucleotide and exposed to UVR (UVR + NMN), and a group treated with polypodium leucotomos and exposed to UVR (UVR + PL). All mice were assigned a unique ID number. Before imaging, the mice were anesthetized with 0.05 mL HypDorm (0.158 mg/mL fentanyl citrate, 5 mg/mL fluanisone, 2.5 mg/mL midazolam). To maintain consistent subgroup sizes of six throughout the study, replacement mice from the respective main groups were introduced if mice died. In such cases, data collection continued from the time of replacement, ensuring complete time series data for each month.

### Compound administration and UVR protocol

Freshly made nicotinamide mononucleotide (Life Powders, Galway, Ireland) was administered to the UVR + NMN group through their drinking water in doses of approximately 600 mg NMN/kg body weight. Once a week, approximately 4.718 g of powdered NMN was weighed and dissolved in 70 mL of tap water. The solution was distributed into seven plastic tubes and stored in the freezer until use. Once a day, a tube was thawed, dissolved into 190 mL tap water, and then given to the UVR + NMN group.

The UVR + PL group received polypodium leucotomos through shelf-stable dry food. 1875 mg PL/kg feed (Laboratorie ORONALYS, Remerschen, Luxembourg) was added to the standard laboratory feed, corresponding to a daily dose of 300 mg PL/kg body weight. Supplemented feed was started two weeks before the initiation of UVR.

The three treatment groups: UVR + NMN, UVR + PL, and UVR control were subjected to controlled UVR exposure thrice weekly: Monday, Wednesday, and Friday. They were irradiated from above in their cages, allowing them to move freely. Each exposure session lasted 21 minutes and 14 seconds, delivering equivalent to 3.5 standard erythema doses using solar-simulated radiation within the UVA and UVB range. The UVR was within wavelengths 280–400 nm, containing 5.9 % UVB emission and 94.1 % UVA emission provided by one UV6 tube (Waldmann, Wheeling, IL, USA) and five Bellarium-S SA-1-12 tubes (Wolff, Stuttgart, Germany) [30]. This dose has previously been used to induce tumor development in hairless mice of this strain [30, 31]. During each exposure session, the UV emission spectrum was measured with two spectroradiometers (Solatell, Sola-Hazard 4D Controls Ltd, Cornwall, UK and Jaz, Ocean Optics, Dunedin, FL, USA) to ensure consistent exposure between sessions. The same procedure was applied to all UVR exposure sessions throughout the study.

### OCT imaging

Volumetric OCT images were acquired using a multi-beam VivoSight Dx (*Michelson Diagnostics Ltd*, Maidstone, Kent, UK) once per month over a period of 7 months. An area of 6 × 6 mm was scanned, acquiring 1355 A-scans per B-scan and 120 B-scans per volume with an A-scan spacing of 4.4 μm. The system used four beams of light to focus on different depths, enhancing the penetration depth. The light source was a swept source with a central wavelength of 1310 nm and a bandwidth of 140 nm. The power at the sample was 5–10**μ*W*. The system used a line-scan rate of 20 kHz, and the resolution was 5.5 μm and 7.5 μm in the axial and lateral directions, respectively. Images were obtained from each mouse’s back, side, and stomach, where small ’x’ tattoos on their back and side marked the imaging locations to ensure imaging of approximately the same area between sessions. These specific areas were chosen because they were exposed to different levels of UVR. Images were acquired by gently folding the mouse skin on top of a finger, on which the system’s probe was held. When imaging the stomach, the mouse was laid on its side for the duration of the scan (approximately 30 seconds).

### Data processing

One volume scan from each imaging location of each mouse was selected for data processing. For each B-scan, a running filter downsized the data by averaging sets of 10 averaged A-scans. The first and last 15 averaged A-scans in each B-scan were removed from the analysis as these typically had low intensities. Within each 6 × 6 mm area, a total of 12,720 averaged A-scans were obtained comprising 106 usable averaged A-scans per B-scan across 120 B-scans per volume. The two metrics, the skin thickness, and the attenuation coefficient were extracted using a custom software developed in MATLAB R2023a (*The MathWorks*, *Inc.*, Natick, Massachusetts, USA).

#### Skin thickness.

The skin thickness, including both the epidermis and the dermis, was measured by identifying the outermost layer and the boundary between the dermis and the underlying muscle. The outermost skin layer was identified as the first peak detected above a predefined noise floor, set to the mean intensity of the air in the A-scan data. The boundary between the dermis and the muscle layer was determined when the A-scan intensity first dropped below the mean intensity of the muscle layer. The mean intensity of the air and the muscle layer was determined by analyzing a set of reference images comprising one dataset from each group at baseline. A.V.V. visually identified the air and muscle regions in each dataset and calculated the mean intensity in this region in each reference image. Additionally, a peak prominence threshold was set to filter out noise and ensure accurate peak detection; this threshold was determined experimentally to balance between detecting true peaks and avoiding noise artifacts. Skin thickness was measured as the distance from the first detected peak to the boundary between the dermis and muscle layer. Measurements were repeated for all 12,720 averaged A-scans within each volume scan, and the mean skin thickness for each volume scan was calculated.

#### The attenuation coefficient.

To determine an approximation of the attenuation coefficient, the single-scattering model $\langle A(z)\rangle \propto A_{0}\;\cdot \;e^{\left(-\mu_{\text{t}}\cdot z\right)}$ was used. The model, based on Lambert-Beer’s law $I(z)=I_{0}\;\cdot \;e^{\left(-\mu_{\text{t}}\cdot z\right)}$, assumes single scattering, forward propagation of light, the absence of noise or instrumental effects, and plane wave illumination [32]. A region of interest (ROI) was defined from the second detected peak, assumed to be the dermal-epidermal junction (DEJ), to the boundary between the dermis and the underlying muscle to focus primarily on the dermis. Since images exported by the VivoSight instrument are saved with a logarithmic intensity scale, a linear fit was performed on the intensity values within the ROI to approximate the attenuation coefficient. This procedure was repeated for all averaged A-scans within each volume scan, and the mean value was calculated to determine an approximated attenuation coefficient for each image.

### Histopathology

Histological data were collected at 16 and 30 weeks to minimize the number of biopsies performed on each mouse. A 4 mm skin biopsy was taken from six mice from each of the main groups to avoid disrupting the OCT-imaging site. The biopsies were taken from non-tumor skin on the lower back of the mice corresponding to the OCT imaging sites to allow for direct comparison between histological and OCT-derived measurements. Biopsies were obtained from the same mice at both time points. All samples were processed similarly: they were fixed in 4% buffered formaldehyde, dehydrated, and embedded in paraffin wax [33]. These were cut into sections of 10 μm and stained with hematoxylin and eosin. Stained sections of the biopsy were imaged at a magnification of 20x using a MoticEasyScan digital slide scanner (Motic, Spain). Three locations in the sample were randomly selected, and epidermal and dermal thickness were quantified at each location using the built-in Motic DSAssistant.

### Pigmentation

Pigmentation scores were assessed visually once per month following a protocol adapted from Hansen *et al*. [34]. The evaluation took place in a dark room, where the mice were individually positioned under a bank of fluorescent long-wave TL08 UVA tubes (Philips, Amsterdam, The Netherlands). Exposure to these long-wave UVA rays caused the pigmentation to be visible in various shades of purple. To classify the pigmentation on the mouse’s back, a Kodak grayscale (Kodak, New York, USA) was used. An unblinded investigator assigned a numerical shade based on the closest match to the Kodak scale.

### Statistical analysis

Statistical analysis was conducted in RStudio using R version 4.2.1 (*R Foundation for Statistical Computing*, Vienna, Austria) [35] with a significance level set at 0.05. For each metric at all three imaging locations, a p-value was calculated using one-way analysis of variance (ANOVA) at baseline and after 7 months of UVR exposure to confirm that the groups were similar at baseline but significantly different after UVR exposure.

Mann-Whitney U tests were subsequently applied only at imaging sites where significant differences were found by ANOVA, to assess differences between the groups. Outliers above or below 3 times the interquartile range (IQR) were removed from the dataset before calculating p-values for the Mann-Whitney U tests. First, tests were performed between groups, comparing each group at baseline with the same group at month 7, and second, the tests were used to compare the control group to the groups receiving UVR (UVR + NMN, UVR + PL, and UVR control) at month 7. Finally, additional Mann-Whitney U tests were used to compare the UVR control group to the groups treated with photo-protective agents (UVR + NMN and UVR + PL). Some mice were replaced during the study due to humane endpoints, and therefore, we used an unpaired test to ensure consistency across the dataset.

For the pigmentation data, which was only measured at the back, Mann-Whitney U tests were used to compare the control group to each of the UVR-exposed groups, as well as the UVR control groups to the two treated groups (UVR + NMN and UVR + PL).

## Results

### Assessment of skin pigmentation and tumor development

**Fig 1:**
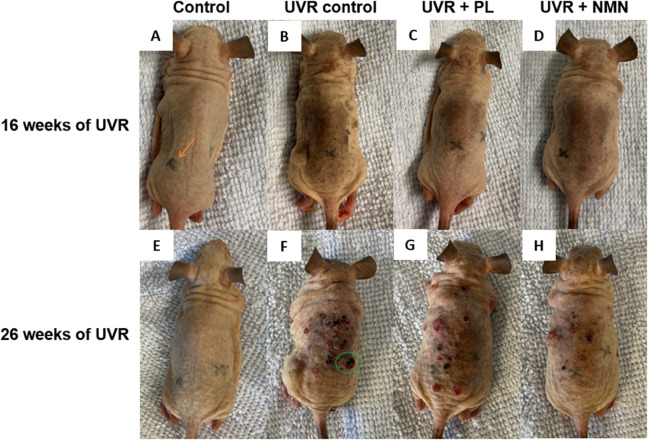
Representative photographs of mice from each group after 16 and 26 weeks of UVR exposure. Tattoos, marking imaging locations (arrow in A), are seen on the back and the side. Images (A) and (E) represent the control group, images (B) and (F) show the UVR control group, images (C) and (G) show the UVR + NMN group, and images (D) and (H) show the UVR + PL group. Images on the top row (A-D) were taken after 16 weeks of UVR exposure, and images on the bottom row (E-H) were taken after 26 weeks of UVR exposure. The progression of skin damage, including SCC development (green circle in F), is evident in images (F-H) after 26 weeks. The same mice were photographed at both time points.

To evaluate the visible photodamage of the skin, photographs were taken throughout the study, as seen in Fig 1. At 16 weeks of UVR exposure, the control group maintained thin, elastic, and smooth skin upon palpation. In contrast, the UVR control group exhibited noticeable pigmentation on the back. The UVR + NMN group and the UVR + PL also showed signs of pigmentation, but visually the extent seemed less severe compared to the UVR control group.

After 26 weeks of UVR exposure, bigger differences between the four groups were observed. The control group continued to show uniform and smooth skin, with no noticeable changes from the 16-week observation. However, the UVR group showed much more skin damage, including the development of SCCs. Tattoos marking the imaging location were no longer easily visible, and upon palpation, the skin felt thicker and more rigid. The UVR + NMN and UVR + PL mice also exhibited signs of apparent photodamage and development of SCCs.

**Fig 2:**
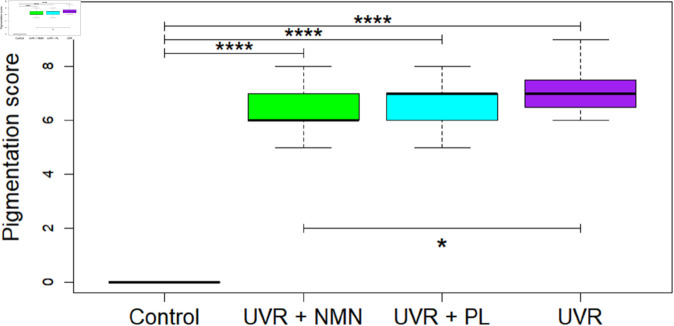
The dorsal pigmentation after 24 weeks is shown as boxplots, n=6 (Control), 22 (UVR + NMN), 17 (UVR + PL), and 11 (UVR Control). The black line within each box represents the median, while the boxes themselves display the interquartile range (IQR). Whiskers extend to maximum and minimum values within 1.5 times the IQR, and outliers are marked as individual dots. Statistical significance was assessed using the Mann–Whitney U test. Asterisks indicate significant differences between groups (*p<0.05; ****p<0.0001). Non-significant comparisons are not shown.

Pigmentation scores obtained from the back location showed significant differences between the control group and all UVR-exposed groups (*p*<0.0001) at 24 weeks, as seen in Fig 2. Furthermore, a significant difference was observed between the UVR control group and the UVR + NMN group (*p* = 0.0128), while no significant differences were found between the UVR control group and the UVR + PL group using a Mann-Whitney U test.

### Histopathology

**Fig 3:**
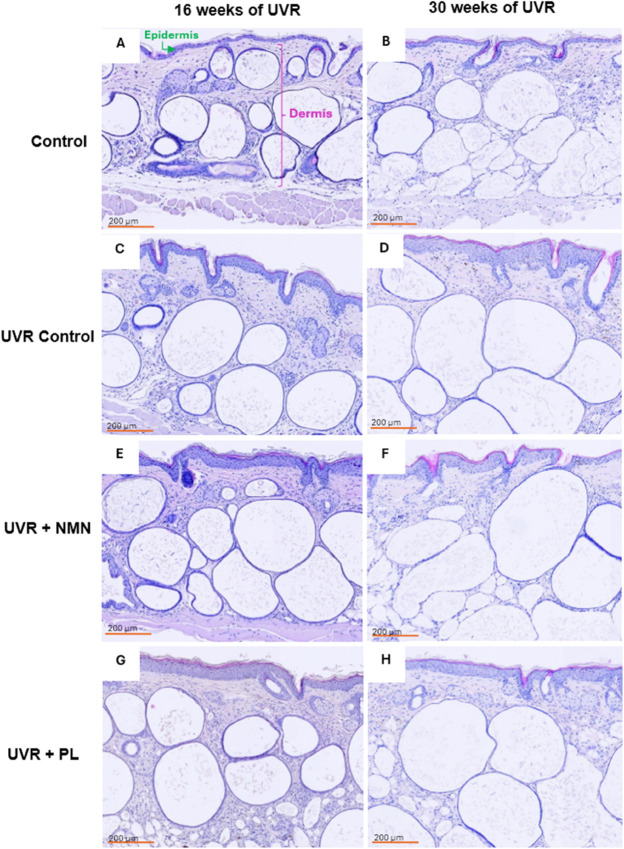
Hematoxylin and eosin-stained biopsies at 16 and 30 weeks of UVR exposure. (A) and (B) the control group, (C) and (D) the UVR control group, (E) and (F) the UVR + NMN group, and (G) and (H) the UVR + PL group. Images on the left (A), (C), (E), and (G) are taken after 16 weeks of UVR exposure, while those on the right (B), (D), (F), and (H) are taken after 30 weeks of UVR exposure. The epidermis and dermis are indicated in (A). Biopsies were taken from the same mice at both time points.

Histopathological assessment of the skin showed notable differences between the control group and each of the UVR-exposed groups. Representative histology images are shown in Fig 3. At 16 weeks, all UVR-exposed groups exhibited similar noticeable epidermal thickening compared to the control group. After 30 weeks of UVR exposure, more pronounced differences between the groups were observed. The control group showed no noticeable changes in skin histology from 16 to 30 weeks. In contrast, the epidermis thickened in the UVR-exposed groups due to keratinocyte proliferation, as seen by the increase in keratinocyte layers [8]. The UVR control group visually exhibited the largest epidermal thickening, while the treated groups did not show substantial differences in epidermal thickness compared to the 16-week observation. Finally, comparing all the images, it was observed that the total skin thickness was greater in the UVR groups, with pockets of adipose tissue taking up larger proportions of the dermal layer. These arise from sebaceous glands that have lost their glandular ducts, leading to the accumulation of fluid and fat in the dermis [36, 37]. The underlying muscle layer remained visible only in the control group after 30 weeks of UVR exposure.

**Fig 4:**
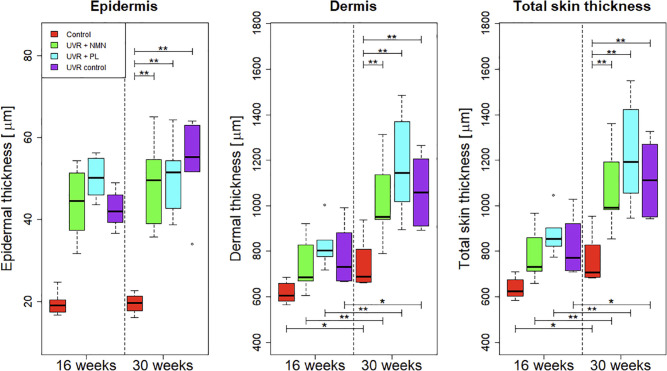
Box plots showing the epidermal, dermal, and total skin thickness measured from hematoxylin and eosin-stained skin sections on the back, across the four groups: control group, UVR control, UVR + NMN, and UVR + PL after 16 and 30 weeks of UVR exposure. The black line within each box represents the median, while the boxes themselves display the interquartile range (IQR). Whiskers extend to maximum and minimum values within 1.5 times the IQR, and outliers are marked as individual dots. The box plots from the total skin thickness are the sum of the epidermal and dermal layers. Statistically significant differences are indicated as (^*^) for *p*<0.05 and (^**^) for *p*<0.01. Asterisks at the bottom indicate differences within the same group between time points, while asterisks at the top indicate differences between each UVR-exposed group and the control group at 30 weeks. Non-significant comparisons are not shown.

Quantitative measurements of the epidermal and dermal thickness are shown as box plots in Fig 4. In general, both epidermal and dermal thickness increased in the UVR-exposed groups compared to the control groups at both 16 and 30 weeks.

To evaluate the overall impact of UVR, the skin thickness was analyzed separately for the epidermal, dermal, and total skin thickness (epidermis + dermis). A one-way ANOVA performed on total skin thickness revealed significant differences between the four groups at both 16 weeks (*p* = 0.004) and 30 weeks (*p* = 0.002). Significant increases in skin thickness over time were observed in all groups for at least one of the layers, with dermal thickness showing the most consistent change (see Table 1). Pairwise comparisons confirmed significantly increased skin thickness in the UVR-exposed groups compared to the control group across all layers. However, no evidence of photoprotective effects was observed for either of the treated groups (see Table 2). Overall, the same trends of UVR-induced thickening and lack of photoprotective effects were consistently observed across the epidermal, dermal, and total skin layers.

**Table 1:** Comparison of skin thickness at 16 and 30 weeks measured from hematoxylin and eosin-stained histological sections. Mann–Whitney U tests were performed to compare skin thickness within each group between the two time points (16 and 30 weeks). Results are shown separately for epidermal, dermal, and total skin thickness. Statistically significant differences are indicated as (^*^) for *p* < 0.05 and (^**^) for *p* < 0.01.

**Epidermal thickness: 16 weeks vs. 30 weeks**				
16 weeks \ 30 weeks	Control	UVR + NMN	UVR + PL	UVR control
Control	0.818			
UVR + NMN		0.485		
UVR + PL			0.818	
UVR control				0.065
**Dermal thickness: 16 weeks vs. 30 weeks**				
16 weeks \ 30 weeks	Control	UVR + NMN	UVR + PL	UVR control
Control	0.0152 (^*^)			
UVR + NMN		0.009 (^**^)		
UVR + PL			0.004 (^**^)	
UVR control				0.0152 (^*^)
**Total skin thickness: 16 weeks vs. 30 weeks**				
16 weeks \ 30 weeks	Control	UVR + NMN	UVR + PL	UVR control
Control	0.015 (^*^)			
UVR + NMN		0.009 (^**^)		
UVR + PL			0.004 (^**^)	
UVR control				0.015 (^*^)

**Table 2:** Pairwise comparisons of epidermal, dermal, and total skin thickness at 30 weeks measured from hematoxylin and eosin-stained histological sections. Mann–Whitney U tests were conducted to compare skin thickness between the control group and the UVR-exposed groups (UVR + NMN, UVR + PL, and UVR control), as well as between the UVR control group and the two treated groups. The results are shown separately for epidermal, dermal, and total skin thickness. Statistically significant differences (*p* < 0.01) are indicated with (^**^).

**Epidermal thickness (30 weeks)**				
	Control	UVR + NMN	UVR + PL	UVR control
Control		0.002 (^**^)	0.002 (^**^)	0.002 (^**^)
UVR control		0.485	0.699	
**Dermal thickness (30 weeks)**				
	Control	UVR + NMN	UVR + PL	UVR control
Control		0.008 (^**^)	0.004 (^**^)	0.015 (^**^)
UVR control		1.00	0.394	
**Total skin thickness (30 weeks)**				
	Control	UVR + NMN	UVR + PL	UVR control
Control		0.004 (^**^)	0.004 (^**^)	0.009 (^**^)
UVR control		1.00	0.394	

### OCT images

**Fig 5:**
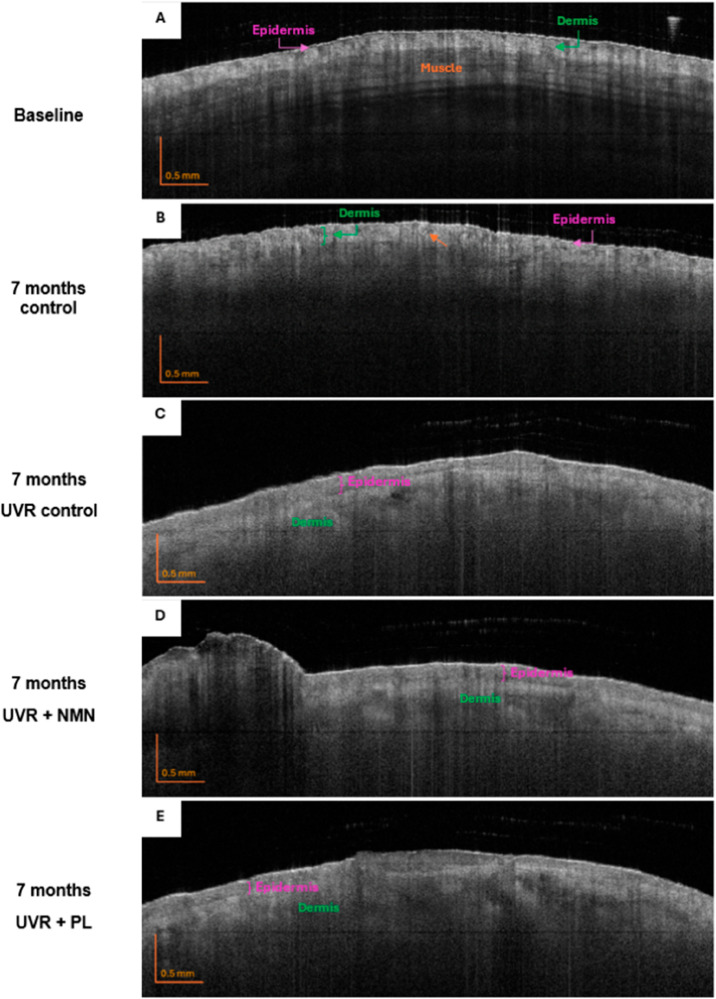
OCT images (B-scans) of the back location at baseline and after 7 months of UVR exposure. (A) Representative baseline image with the epidermis, dermis, and muscle layers indicated. (B-E) Images after 7 months of exposure for different groups: (B) control, (C) UVR control, (D) UVR + NMN, and (E) UVR + PL. The arrow points to the inclusion of adipose tissue, which is evident from the inhomogeneous scattering observed within the dermal layer.

Qualitative assessments of the OCT B-scans showed differences between the control group and the UVR-exposed groups. Representative B-scans are shown in Fig 5. At baseline, all mice had similar skin structures, as no mice had received any UVR exposure. At baseline, the layers were well-defined into the epidermis, the dermis, and the underlying muscle. After 7 months, the control group remained similar to the baseline, but with reduced contrast due to skin aging. In both the baseline and the 7-month control images, the layers were distinct, and it was possible to identify the adipose tissue, which appears as big white cells in the histology images, Fig 3, and as inclusions in the dermis in the OCT-images (marked by the orange arrow).

In contrast, the UVR-exposed groups show less contrast with less defined layers compared to the baseline and 7 months control. Furthermore, UVR groups have a darker appearance of the dermis, and a thickening of both the epidermis and dermis is visible, aligning with the observations from the histology. However, minimal differences can be observed among the UVR-exposed groups.

### OCT-derived skin thickness

**Fig 6:**
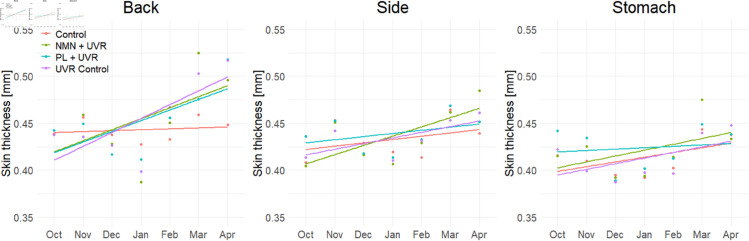
Development of the skin thickness at the back, the side, and the stomach for the four groups: control group, UVR control, UVR + NMN, and UVR + PL. Dots represent the mean skin thickness within each group each month, and the lines depict the overall trend for each group.

A longitudinal overview of the measured skin thickness is shown in Fig 6, separated by group and imaging location. At the back, there is a noticeable increase in skin thickness over time in all groups, with the largest increase observed in the UVR-exposed groups. A similar trend of increasing skin thickness over time was observed in the UVR-exposed groups at the side and stomach locations, with the back showing the greatest increase, the side showing a moderate increase, and the stomach showing the least increase. This pattern corresponds with the varying levels of UVR exposure, as the stomach is more shielded compared to the back.

One-way ANOVA was performed separately at baseline and again at month 7 to compare skin thickness among the groups at each imaging site. No significant differences were found among the groups at baseline at the back (*p* = 0.994), the side (*p* = 0.305), or the stomach (*p* = 0.125). However, after 7 months of UVR exposure, a significant difference was observed among the groups at the back (*p* = 0.024), while no differences were found at the side (*p* = 0.612) or the stomach (*p* = 0.975). Due to the significant differences observed only at the back location, subsequent comparisons focus solely on this imaging site.

Additionally, comparing each group at baseline and after 7 months using Mann-Whitney tests, no significant difference was observed for the control group, while significant differences were observed for each of the UVR-treated groups, see Table 3.

**Table 3:** Comparison of OCT-derived total skin thickness at baseline and at 7 months. Total skin thickness was measured using OCT at baseline and at 7 months to assess changes over time. The Mann-Whitney U test was performed to compare total skin thickness within each group between the two time points. Statistically significant differences are indicated as (^*^) for *p* < 0.05 and (^**^) for *p* < 0.01.

Baseline \ Month 7	Control	UVR + NMN	UVR + PL	UVR control
Control	0.247			
UVR + NMN		0.008 (^**^)		
UVR + PL			0.030 (^*^)	
UVR control				0.019 (^*^)

**Fig 7:**
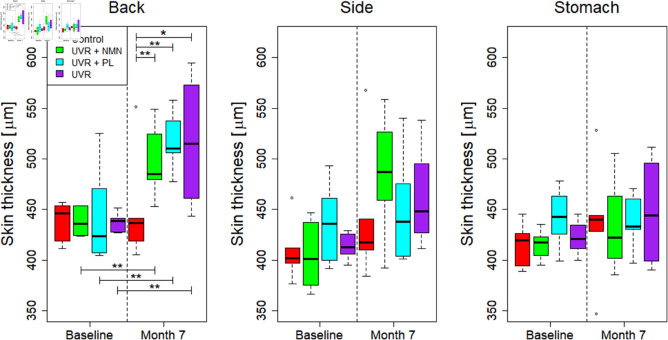
Box plots showing the OCT-derived skin thickness measured on the back, side, and stomach: control group, UVR control, UVR + NMN, and UVR + PL. The black line within each box represents the median, while the boxes themselves display the interquartile range (IQR). Whiskers extend to maximum and minimum values within 1.5 times the IQR, and outliers are marked as individual dots. Comparisons are made between baseline and after 7 months of UVR exposure to assess changes in the skin thickness. Statistically significant differences are indicated as (^*^) for *p* < 0.05 and (^**^) for *p* < 0.01. Non-significant comparisons are not shown.

Finally, at month 7, significant differences were observed between the control group and each of the UVR-treated groups, but no significant differences were observed between the UVR control and the treatment groups, see Table 4. These results are visualized in Fig 7 and are consistent with the trends from the histological analysis of total skin thickness.

**Table 4:** Pairwise comparisons of OCT-derived total skin thickness. Mann-Whitney U tests were performed to compare OCT-derived total skin thickness between the control and UVR-exposed groups, as well as between the UVR control and treated groups (UVR + NMN, UVR + PL) at month 7.

	Control	UVR + NMN	UVR + PL	UVR control
Control		0.004 (^**^)	0.008 (^**^)	0.016 (^*^)
UVR control		0.914	1.00	

### The attenuation coefficient

**Fig 8:**
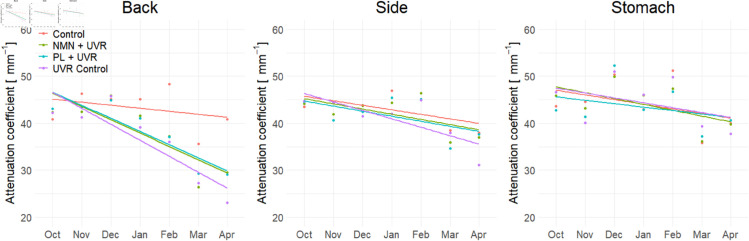
Development of the attenuation coefficient $\mu_{\text{OCT}}$ at the back, the side, and the stomach for the four groups: control group, UVR control, UVR + NMN, and UVR + PL. Dots represent the mean attenuation coefficient within each group each month, and the lines depict the overall trend for each group.

Similar to the skin thickness derived from OCT, a longitudinal overview of the measured attenuation coefficients is shown in Fig 8. In general, the attenuation coefficient is observed to decrease over time. The biggest differences between the four groups can be observed on the back. The UVR groups have the steepest decrease, while the control group only shows a slight decrease over time. The same trend is observed on the side, although the decrease is less steep compared to the back. At the stomach, the attenuation coefficient is slightly decreasing, with all four groups being very similar. Interestingly, the UVR control group appears to demonstrate the biggest decrease for both the back and side imaging locations.

One-way ANOVA was used to compare the attenuation coefficients among the four groups at both baseline and after 7 months. There were no significant differences in the attenuation coefficient at the back (*p* = 0.475), the side (*p* = 0.943), or the stomach (*p* = 0.199) at baseline. However, after 7 months of UVR exposure, a significant difference in attenuation coefficients was observed between the groups at the back location (*p* < 0.001), while no significant differences between the groups was found at the side (*p* = 0.472) or the stomach (*p* = 0.895).

To assess changes over time, Mann-Whitney U tests were performed within each group, comparing baseline to month 7. No significant change was observed for the control group, but significant differences were observed for each of the UVR-treated groups, see Table 5.

**Table 5:** Comparison of OCT-derived attenuation coefficient at baseline and at 7 months. The attenuation coefficient was measured at baseline and at 7 months to assess changes over time. The Mann-Whitney U test was performed to compare the attenuation coefficient within each group between the two time points. Statistically significant differences are indicated as (^**^) for *p* < 0.01.

Baseline \ Month 7	Control	UVR + NMN	UVR + PL	UVR control
Control	0.589			
UVR + NMN		0.002 (^**^)		
UVR + PL			0.009 (^**^)	
UVR control				0.009 (^**^)

**Fig 9:**
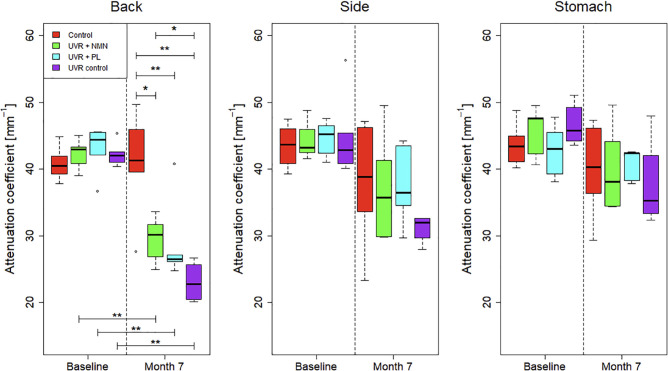
Box plots showing the approximated attenuation coefficient $\mu_{\text{OCT}}$ measured on the back, side, and stomach across the four groups: control group, UVR control, UVR + NMN, and UVR + PL. The black line within each box represents the median, while the boxes themselves display the interquartile range (IQR). Whiskers extend to maximum and minimum values within 1.5 times the IQR, and outliers are marked as individual dots. Comparisons are made between baseline and after 7 months of UVR exposure to assess changes in the attenuation coefficient. Statistically significant differences are indicated as (^*^) for *p* < 0.05 and (^**^) for *p* < 0.01. Non-significant comparisons are not shown.

Furthermore, at month 7, Mann-Whitney tests were conducted to compare the control group with each of the UVR-treated groups, all of which showed significant differences. Additional comparisons at month 7 revealed a significant difference between the UVR control group and the UVR + NMN, whereas the comparison between the UVR control group and the UVR + PL group did not reach statistical significance. These results are visualized in Fig 9 and p-values are summarized in Tables 5 and 6.

**Table 6:** Pairwise comparisons of attenuation coefficient at 7 months. Mann-Whitney U tests were performed to compare attenuation coefficients between the control and UVR-exposed groups. Additionally, comparisons were made between the UVR control group and the treated groups (UVR + NMN, UVR + PL) to assess potential treatment effects. Statistically significant differences are indicated as (^*^) for p<0.05 and (^**^) for *p* < 0.01.

	Control	UVR + NMN	UVR + PL	UVR control
Control		0.026 (^*^)	0.009 (^**^)	0.009 (^**^)
UVR control		0.019 (^*^)	0.200	

## Discussion

In this study, it was observed that repeated exposure to UVR over time significantly increased the skin thickness measured by OCT, and decreased the OCT-derived attenuation coefficient at the back location of the mice. As shown in Figs 6 and 8, the UVR control group exhibited the most substantial changes in both metrics, followed by the two treated groups, and only minor changes to the control group.

The aim was to investigate whether longitudinal trends in skin response to UVR could be detected using non-invasive OCT-based measurements. We initially assumed a linear progression over time, consistent with the expected accumulation of photodamage and aging. However, our results suggest that this relationship is likely more complex, and a linear approximation may be too simplistic to fully capture the biological response. Nevertheless, the overall direction of change remains consistent with UVR-induced damage.

Treatment with NMN resulted in a significant change in attenuation coefficient compared to the UVR group, mirroring the effect observed for pigmentation. This could indicate a subtle effect of photoprotection that should be explored in further studies.

The observed extent of photodamage varied by the imaging location, with the most photodamage observed on the back location. This observation aligns with the irradiation setup, where the mice were exposed from above, resulting in the highest exposure on the back and the lowest exposure on their stomach.

Two extreme outliers were removed from the analysis: one from the control group (OCT-derived skin thickness) at month 7, and one from the UVR + PL group at month 7 (attenuation coefficient). In the scan used to measure skin thickness, the placement of the focal plane was much closer to the skin surface, leading to an elevated mean intensity level of the muscle layer compared to the other scans. This deviation may have impacted the detected boundary of the muscle layer since the algorithm we used was based on a global threshold. In the scan used to measure the attenuation coefficient, all other scans from this group showed numerous SCCs, but this specific scan did not have any visible tumors. The higher attenuation coefficient observed in this outlier may be since this particular location incurred less photodamage than other nearby areas, resulting in a higher attenuation coefficient. In this study, we included six mice per group for OCT imaging, which provided useful initial findings. However, increasing the sample size in future studies may help to reveal statistically significant trends that were not observed here.

### Skin thickness

The algorithm used in our study was designed to measure the combined thickness of the epidermis and dermis. This approach was chosen as, with an axial resolution of 5.5 μm and an epidermal thickness around 10 μm in the control group [38], the algorithm’s fit would only rely on 2-4 data points, reducing its sensitivity.

The algorithm’s quantified measurements of the skin thickness indicated a progressive increase over time in response to acute photodamage. This trend was verified by the histology images from Figs 3 and 4, which visually confirmed both epidermal and dermal thickening in the UVR-exposed groups after 16 and 30 weeks of UVR exposure.

A closer look at the histology images, reveals that the pockets of adipose tissue in the dermal layer of the UVR control group are generally larger and occupy a greater proportion of the dermal layer. Future studies could explore this observation further by quantitatively assessing the proportion and distribution of adipose tissue within the dermal layer to better understand potential UVR-induced remodeling and its role in photodamage progression. It is unclear whether the thickening of the dermis was a direct response to the UVR exposure or whether inherent biological variation could be a confounding factor. Some studies have indicated that the dermal thickness may change in response to UVR exposure [8]. Furthermore, we observed similar trends for both epidermal and dermal thickness from histology and OCT-measured skin thickness, with significant p-values appearing at the same measurement points, suggesting that our approach is well correlated with the histological assessment. Since dermal changes in response to UVR are not well-documented in the literature, further studies should verify the effect of UVR exposure on dermal thickness.

It should be noted that the dermal thickness showed considerable variability across the histological sections (Figs 3 and 4). This variation may be influenced by factors such as resection depth, the choice of measurement points, anatomical location, or inherent biological differences between animals. These elements can introduce uncertainty into histological quantification and may affect the precision of the histological reference. This could explain the difference in dermal thickness between the histology and OCT images. Nonetheless, we were still able to see significant differences between the groups, and the trend remains unchanged.

One potential limitation of the study involves the method used to measure the OCT-derived skin thickness. In the UVR-exposed groups, the boundary between the dermis and muscle layer becomes less clearly defined as photodamage increases. This could be due to changes in the scattering properties of the dermis, making its optical properties more similar to the underlying muscle layer. As a result, the edge detection, which relied on the mean pixel intensity in the muscle layer to set a noise floor, may have been a source of variability when defining the boundary. Future studies could benefit from the use of deep learning algorithms to improve boundary segmentation. Additionally, incorporating adaptive thresholding may help account for local variations in signal intensity, particularly in scans with elevated noise or atypical morphology. A higher resolution OCT system could further enhance the differentiation of skin layers at later time points. However, our overall approach was systematic and still indicates trends in skin thickening across groups consistent with histopathological assessment.

### The attenuation coefficient

Prior to the study, the feasibility of using an approximated attenuation coefficient to evaluate photodamage in mouse skin was uncertain. We hypothesized that the attenuation coefficient would increase due to enhanced scattering from more pigmentation in the skin as a response to UVR exposure [39, 40]. Contrary to this expectation, the results demonstrated a decrease in the attenuation coefficient. One possible explanation is that attenuation may have increased significantly within the epidermal layer, resulting in less light penetrating the dermis, and leading to an overall apparent decrease in attenuation over the entire measured region. Additionally, the proliferation of keratinocytes contributes to the overall skin’s thickening and changed scattering properties, as it reduces the signal [8]. This interpretation aligns with the observed darkening of the dermal layer in UVR-exposed groups as seen in Fig 5.

Multiple methods have been used to extract $\mu_{OCT}$ and the scattering coefficient, $\mu_{s}$, from OCT images. One potential limitation of the study is that the model we employed to extract the attenuation coefficient assumes that only ballistic scattering occurs within the sample. However, studies have shown that in highly forward-scattering media, multiple scattering significantly contributes to the OCT signal intensity [41–43]. Additionally, the model we used does not account for absorption. Given that more natural light-absorbing chromophores such as melanin are introduced in photodamaged tissue, it may lead to increased absorption [40, 44]. Although melanin absorption is minimal in the wavelength range used for our measurements (around 1300 nm) and is often neglected, this can still impact the accuracy of the measured attenuation coefficients. Overall, while the single scattering model that we used may not measure the exact attenuation coefficient, it is well-suited for identifying trends in tissue changes over time, which was the primary goal for this study [22]. Additionally, considering the relatively thin skin of mice, the chosen approach should be adequate. In future studies, we might consider using a model that incorporates multiple scattering and absorption effects, such as the extended Huygens-Fresnel model (EHF) [41, 45] to improve the accuracy of the extracted attenuation coefficients, especially when investigating higher-scattering tissue. Moreover, employing depth-resolved or piecewise fitting of the attenuation could enable layer-specific analysis of optical properties, particularly useful for separating changes in the epidermis and dermis. This would be especially relevant in studies using higher-resolution OCT systems or advanced segmentation approaches [46].

Very few studies have reported measurements of the OCT-derived attenuation coefficient in skin. Turani *et al*. utilized the EHF to estimate the scattering and absorption coefficients, and scattering anisotropy, reporting scattering coefficients <10 ${\text{mm}}^{-1}$ for nearby healthy skin and >20 ${\text{mm}}^{-1}$ for melanomas in human skin [26]. In contrast, the OCT-derived attenuation coefficient $\mu_{OCT}$ measured in our study ranged from 38–45 ${mm}^{-1}$ at baseline and 20–33 ${mm}^{-1}$ for the UVR groups after 7 months. This discrepancy is most likely due to anatomical differences between human and mouse skin, as well as the use of the specific attenuation models. For example, the mouse dermis contains pockets of adipose tissue, which may increase the scattering coefficient, Additionally, our measurements focused on the attenuation properties of the dermis, while Turani *et al*. [26] measured primarily the epidermis. Finally, while Turani *et al*. employed the EHF model to account for both single and multiple scattering, our study applied a model which only accounts for single scattering. Although this simplification might reduce the accuracy of the absolute attenuation coefficients, the main focus of our study was to investigate trends across the groups. By maintaining a systematic approach to attenuation coefficient extraction and ensuring uniform application of the method to all images, the observed trends remain robust, even if the values may not be directly comparable. This justifies our use of the single scattering model despite the presence of multiple scattering.

### The efficacy of NMN and PL as photodamage prevention agents

There are multiple ways to monitor photodamage. In this study, we chose to employ two metrics derived from OCT images to evaluate the efficacy of NMN and PL in mitigating UVR-induced tumor development. Although our study did not find significant differences in tumor onset, number, size, or growth when evaluating these treatments (results not shown) [47], we did find that the median pigmentation was significantly lower in the UVR + NMN group compared to the UVR group. Pigmentation is induced in response to UVR and is believed to prevent subsequent damage. This protective mechanism may have contributed to the observed difference in attenuation, as the OCT-derived attenuation coefficient revealed the same significant difference between the UVR control group and the UVR + NMN group. It may be the case that the difference in attenuation reflects not only the difference in pigmentation but potentially also reveals other sub-resolution alterations in the tissue structure, such as variations in cell shape and size, although further investigation is required to validate this. The fact that the OCT-derived metrics detected subtle changes that were not apparent through traditional tumor assessment suggests that OCT may have the potential as a method for detecting early photodamage. However, the correlation of these results with biological changes should be further investigated in a future study.

Furthermore, our key findings from the OCT-derived metrics mirrored the trends observed in histological analysis. The significant increase in OCT-derived skin thickness in the UVR-exposed groups was reflected by a corresponding thickening in the histological measurements. Likewise, the attenuation coefficient differed significantly between the UVR control and UVR + NMN groups. This observation aligns with differences seen in pigmentation scores, suggesting a biological basis for this contrast. While the histological analysis did not detect treatment effects for NMN or PL in terms of total skin thickness, the observed consistency between UVR-induced changes captured by both OCT and histology supports the potential of OCT as a non-invasive tool for assessing early photodamage.

Since the photoprotective treatments were administered orally, factors such as absorption, metabolism, and compound stability may have influenced the bio-viability and ultimately the treatment efficacy. While these pharmacokinetic variables were not assessed in this study, they could contribute to the inter-animal variability and should be considered in future studies. To improve the stability of the NMN compound, fresh prepared solutions were made prior to the administration. For the PL treatment, a dry feed containing the specified amount of extract was used, which is considered shelf-stable and allows for consistent dosing.

Future work could benefit from repeating the study with a well-established photoprotection such as sunscreen with high sun protection factor (SPF), to further validate the use of OCT-derived skin thickness and $\mu_{OCT}$ to evaluate photodamage prevention.

## Conclusion

This study demonstrates that OCT can be effectively used for real-time, non-invasive monitoring of photodamage, offering a potential method for longitudinally assessing the efficacy of photodamage prevention treatments. Notably, the p-values from our chosen OCT-derived metrics: skin thickness and attenuation coefficient closely mirrored the epidermal thickness measurements obtained from histology, indicating a strong alignment with these invasive ground truth findings. This suggests that OCT can provide equivalent information to histology images without the need for invasive procedures. While other methods, such as skin thickness or epidermal thickness (measured via histology) did not show a significant effect of the use of the NMN or PL, the attenuation coefficient suggests that NMN potentially may have mitigated some effects of the UVR. Taken together, our results highlight OCT’s potential as a screening tool for assessing photodamage. Given the rising incidence of skin cancer, particularly SCC, OCT offers a promising non-invasive approach for improving both early detection and prevention strategies.
